# Combatting the misuse of benzodiazepines and related Z drugs in French general practice: a clinical review

**DOI:** 10.3399/bjgpopen20X101014

**Published:** 2020-03-04

**Authors:** Gaetan Gentile, Maryse Lapeyre-Mestre, Joelle Micallef

**Affiliations:** 1 Professor, Département Universitaire de Médecine Générale, Faculté des Sciences Médicales et Paramédicales, Aix-Marseille Université, Marseille, France; 2 Research Professor, Institut des Neurosciences des Systèmes, Aix-Marseille Université, Marseille, France; 3 Lecturer, Département de Pharmacologie Médicale et Clinique, Center Hospitalier Universitaire de Toulouse, Université de Toulouse, Toulouse, France; 4 Professor, Department of Clinical Pharmacology and Pharmacovigilance, Centre d'Addictovigilance PACA Corse, Timone Hospital, Marseille, France; 5 Research Professor, Institut des Neurosciences des Systèmes, Aix-Marseille Université, Marseille, France

**Keywords:** Prescribing benzodiazepine, General practice, Drug and substance abuse

## Introduction

Benzodiazepines (BZDs) are widely used as anxiolytic, hypnotic, and antiepileptic drugs. They have a fast pharmacodynamic tolerance to hypnotic and anxiolytic effects and long-term prescribing of them is ineffective and potentially dangerous because of the induced dependence. Numerous studies have outlined the high level of their use in Europe, with a particular high level of use in France. The risk of death is not negligible in any patient population,^1^ and can be a result of long-term exposure, acute exposure, or sudden dosage increase.

This article reviews the determinants of BZD prescription by GPs and existing measures to reduce it. A synthesis narrative review of recent published or unpublished international literature on BZD prescription in general practice has been used.

## BZDs in France

### France’s position in BZD consumption

France is second to Spain in the use of anxiolytic BZDs and Sweden and Norway have the highest level of use of hypnotic BZDs, with France in third place. A moderate decrease in BZD use has been observed in France over the past 3 years, but the number of French people consuming BZDs is still too high and many campaigns in France to reduce BZD use have been unsuccessful. In most European countries included in the Agence National de Sécurité du Médicament et des Produits de Santé (ANSM) report,^2^ long-term BZD use is stable over time even though short-term use decreases.^3^

### BZDs consumption and the public health problem

In France, BZDs and related Z drugs (such as zolpidem and zopiclone) are actively monitored by the ANSM ‘addictovigilance’ network, through national survey databases, spontaneous reports by health professionals, and health insurance reimbursement databases.^4^

BZDs are legitimately obtained in pharmacies by GP prescriptions, but are also obtained through ‘doctor shopping’ and through illegal means.^5,6^ The benefit–risk ratio of a long-term treatment is unfavourable. After a few weeks, there is an increased risk of side effects such as daytime sleepiness, memory problems, dependence,^7^ falls and fractures in older patients,^8^ road accidents,^9^ and deaths.^10^ The risks of direct^10,11^ and indirect deaths with the use of BZDs in France (including road safety), pose a public health problem.

Regarding French consumption, urban sales data indicate that 64.6 million boxes of anxiolytic BZDs were sold in 2015 (64.9 million in 2010) and 46.1 million boxes of hypnotics (48.2 million in 2010).^2^ In 2015, the prevalence of use of anxiolytic or hypnotic BZDs was higher among women (16.6%) than men (9.7%), regardless of age. This prevalence increases with age and is highest in women aged ≥80 years (38.3%).^2^

Two means of BZD exposure are medical use and non-medical use,^12^ and several subgroups of BZD use are identified in general practice. In particular, patients with a high prevalence of mental health problems are high consumers of BZDs.^7,13–21^

## Relevance to general practice

### BZD prescription in France

In 1991, recommended dosage and intake conditions (a maximum prescription period of 4 weeks for hypnotics and 12 weeks for anxiolytics^22^) was introduced. The tapering of hypnotic effects after a few days, and anxiolytic effects after a few weeks, is the reason for pharmacological tolerance. The duration of treatment includes the period of weaning off in a stepwise manner; hence the need already underlined in 2007 by the High Authority for Health (HAS) — the independent public authority that contributes to the regulation of the health system through quality — to have a therapeutic contract when the BZD is first prescribed.^23^

GPs do not always adhere to these recommendations, for several reasons found in the studies summarised in Table 1. According to the HAS, GPs do not oppose drug substitution treatments when stopping BZDs, and agree that no long-term BZD treatments should be stopped abruptly, however the reality is more complicated.^24^ The GP should reassess and discontinue treatment by gradual dose reduction to allow BZD weaning.

**Table 1.** Theories on the determinants of benzodiazepines prescription by GPs in France

**Authors / Date / Method / Link**	**Title**	**Main results**
Zaknoun / 2017 /Qualitative studyhttp://www.sudoc.fr/201709651	Stopping benzodiazepines and related long-term prescriptions: difficulties in general practice	Dependence and social difficulties of the patients. Difficulties with drugs detoxication. Non-use of non-drug treatments
Kouakoua / 2017 /Qualitative studyhttp://www.sudoc.fr/225424010	Re-prescription benzodiazepines and related in general practice in the Lille region (France)	Age of the patient. Coprescription. Duration of follow-up of the patients. Social factors: unemployment and precariousness. No use of diagnostic tools
Duboy / 2015 /Qualitative studyhttp://www.sudoc.fr/189878681	Over-consumption of anxiolytic and hypnotic benzodiazepines in France: prospects for improvement of prescribing and withdrawal in general practice	Trivial prescription (empathic prescription). Feeling helpless. ‘Miracle drug’ concept.
Faurie / 2014 /Quantitative studyhttp://www.sudoc.fr/184049601	Determinants of benzodiazepine and related turnover beyond three months: one quantitative study of 187 GPs in Poitou-Charentes (France)	Not satisfied with initial medical training and continuing vocational training. Incomplete professional skills. Time constraints. Patient requirements. Lack of therapeutic alternatives. Lack of interprofessional coordination. Optimistic vision of the drug. Perception of social pressure or increased psychosocial problems

### Measures to reduce BZD misuse in France

GPs are on the front line to find operational solutions to combat BZD misuse. The main principles of good use of BZDs and their deprescribing rarely come from general practice, and the applicability of these principles to the GP–patient relationship is a significant challenge. In France, a prescription for narcotics (such as buprenorphine) and morphine derivatives must be written on a tamper-resistant prescription form, with specific technical particularities; namely, that the dosage and daily dose of medicines must be written out entirely in letters. These prescription forms are also mandatory for some BZDs and Z drugs (zolpidem).^25,26^ In 2011, a pay-for-performance intervention between the Federation of the French GPs and the French Ministry of Health was implemented in order to motivate GPs to improve their practices, but the proportion of patients who continued their BZDs did not decrease. In 2015, the French Ministry of Health decided to decrease the reimbursement of the BZDs from 65% to 15%, but this measure did not reduce the long-term consumption of BZDs (>90% of the French population has supplementary insurance that usually covers the entire copayment).^27^

### Examples of misuse in vulnerable French populations

Examples of patients with BZD consumption are numerous. They often present psychiatric comorbidities, and have vulnerability to increased addiction risk.

French prisoner patients widely consume BZDs, with some prisoners on pre-existing BZD treatment. Prison is an environment that exacerbates anxiety and sleep disorders, and there is a high prevalence of mental health problems.^13^ Work-related psychosocial factors also favour an excess consumption of BZDs and other psychoactive substances.^14^ Older patients are more sensitive to the potential adverse effects of BZDs.^15^ In subjects with Alzheimer’s disease and related diseases, a high consumption of antipsychotics was found. These patients have a greater dependence on care systems, increased hospitalisation rates, and are at greater risk of overprescribing, polyconsumption, and worsening vulnerability.^16^ For older patients, BZD dependence is caused by the duration of treatment, the dose administered, the history of addiction, and the association with the consumption of alcohol. BZD use increases with multiple comorbidities, painful life events, social isolation and/or loneliness at home, hospitalisations, and polypharmacy. The impact of BZD long-term use on cognitive decline and dementia has been the subject of many studies, and the role of BZDs in the onset of dementia remains unknown.^7^

The patients on opioid substitution treatments (OST) have addictive behaviours, and often present psychiatric comorbidities found in epidemiological investigations.^21^ The effects of psychoactive drugs or combinations with other opioids are likely to be sought to cope with withdrawal difficulties or cravings, and patients may thus resort to polypharmacy and ‘medical nomadism’. This could be an explanation for the misuse of buprenorphine or other drugs.^17^ The importance of precise analysis about consumption from the outset, through a urine drug screening test, motivated a cluster randomised study (the ESUB-MG study) which is currently in progress.^18^

Adolescence is a period that brings about a mental health vulnerability with family, relational, and existential conflicts. Use of diverted drugs in the adolescent population mainly concerns anxiolytics and opioid analgesics, often taken in combination with alcohol during occasional experimentation with peers or following a medical prescription.^19^

Patients with psychiatric comorbidities can present complex personalities, which are likely to induce therapeutic ruptures with their usual prescriber, successive failures, or the use of other psychoactive substances. They are particularly exposed to BZDs, and these drugs are frequently misused in terms of duration, dose, and number of concomitant medications.^20^ The vulnerability that comes with psychiatric disorders encourages the use and misuse of BZDs.

### What is proposed and/or should be done in France

#### BZD misuse and the French Addictovigilance Network (FAN)

Reporting cases of dependence or misuse to the FAN is mandatory. It is one of the means of identification among the pharmacoepidemiological surveys used and in which GPs participate. GPs, providing their scientific knowledge, must be encouraged to take part in research activities performed by the FAN contributing pharmacological and methodological experiences. Spontaneous reporting is notoriously insufficient in this field, despite efforts periodically made to promote the importance of spontaneously reporting all suspected cases of misuse through regional or national bulletins, scientific publications, medical meetings, and health professional training.^28^ The actions of the FAN resulted in the withdrawal of flunitrazepam in 2013,^29^ and in the mandating of the tamper‐resistant prescription form for prescribing and dispensing clonazepam and zolpidem with a 28-day duration limit.^25,26^

#### Deprescribing by GPs and withdrawal of BZDs

##### Training and research in general practice

Initial and continuing vocational training, and research are key points to optimise these actions. In order to develop addictovigilance education over the past 15 years, changes in teaching in medical schools have occurred. Training in addictology has appeared since 2011 in medical training, in the first year of common studies in health and during the national examination of the medical internship. Diploma courses on addictology are offered in faculties of medicine. Finally, the reform of general practice in 2016 places addictology in the transversal-specific training at the end of the internship programme for future GPs.

Research in French general practice is organised around learned societies and university general practice departments, in partnership with research orgnisations including Institut National de la Santé et de la Recherche Médicale (INSERM) and Centre National de la Recherche Scientifique (CNRS). The National College of GPs Teachers (CNGE), the College of General Practice (CMG), and other scientific societies promote the research in general practice.

Finally, the discipline of general practice developed its own university pathway with the creation of the Diploma of Specialised Studies in General Practice in 2004, and created the positions of ‘clinical leaders’ involved in research and grouped together in French association of young researchers (FAYR-GP). At present in France, 150 clinical leaders in general practice are appointed to faculties of medicine.

##### Guideline adherence

During the consultation, intense pressure from a patient can feel like blackmail, especially with the threat of changing doctor or consulting another doctor. The GP can either follow the prescribing guidelines, or give in to patient demands and prescribe BZDs.

An interesting approach from the literature would be to limit both the indications and the duration of treatments from the outset.^30^ Unfortunately, these recommendations, which are professional tools, are not always applicable in real life. To overcome this failure, several approaches are proposed in experimental studies (Table 2). Online tutorials and easy-to-use tools in prescribing BZDs need to be developed for use in general practice. Continuing training of GPs and patient education are also to be promoted.

**Table 2.** Theories on other proposed measures to reduce or stop benzodiazepines prescriptions by GPs

**Authors / Date / Method / Link**	**Title**	**Main results**
Berthes / 2013 /Systematic review of the literaturehttp://www.sudoc.fr/172070163	Guide to the first prescription of benzodiazepines in anxiety disorders and insomnia	Interest in practical guides for doctors (indications and conditions of use)
Coispeau / 2016 /Qualitative studyhttp://www.sudoc.fr/192757555	Development of a ESBI-type online training tool for the optimisation of benzodiazepine prescriptions in general practice	Using a ESBI Tutorial ‘Benzos‘ Online Training for GPs
Bullo-Masuyer / 2014 / Qualitative studyhttp://www.sudoc.fr/183108221	Withdrawal of benzodiazepines: can the substitute general practitioner help? Qualitative study in the south region (France)	Interest of the singular conference in the benzodiazepines detoxification. Fight against the therapeutic inertia of the replaced doctor. The substitute modifies the behaviour of the patient but also that of the replaced doctor
Demouveaux / 2017 / Qualitative studyhttp://www.sudoc.fr/221278788	Benzodiazepine withdrawal of elderly patients in general practice: utopia or reality?	Prevention and therapeutic education, improving access to psychotherapy and public involvement in the fight against BZD consumption among older patients

BZDs = benzodiazepines. ESBI = Early Spotting and Brief Intervention.

##### Collaboration and multidisciplinary teams

The reassessment of BZDs prescription can be performed by the GP alone, in a multidisciplinary team, or during hospitalisation with medication reconciliation. Since 2018, pharmacists in France have been authorised to perform shared medication reviews in patients aged >65 years, in cooperation with GPs. Nurses, as part of the 'ASALEE' programme introduced in 2015, may also intervene in weaning in cooperation with GPs.

### Other proposed approaches for optimising the effective use of BZDs

All BZD prescriptions should be written on a tamper‐resistant prescription form, with respect for duration as per the guidelines, and should be non-renewable. The current packaging of drugs could be reviewed using a packaging and a delivery pay-per-view. The BZD prescription refill should be carefully assessed and monitored in terms of dose, duration, efficacy, and adverse events, and may lead to stopping or to switching to another type of management: it must be defined as a ‘re-evaluation order‘.

Other solutions have been suggested in the literature: a conference in the BZD detoxification; the fight against the therapeutic inertia of the ‘replaced’ GP; prevention and therapeutic education, improved access to psychotherapy, and public involvement; a substitute GP modifying the behaviour of the patient, but also that of the replaced GP (Table 2); addiction and weaning managed by motivational interviews and non-drug alternatives; and intervention in older patients in nursing homes.^31^

### Relevance to primary care in other countries

The issue of BZD misuse is one of great magnitude and persistence, but it is not a solely French phenomenon.

In the UK, 7.7% of responders admit misuse, which is comparable with the US.^32^ In January 2018, the UK Department of Health commissioned an evidence review of dependence on prescribed drugs.^33^ In Belgium, the impact of an e-intervention on determinants of BZD prescribing among GPs in vocational training was found to be significant.^34^ In Portugal, a cross-sectional, observational study showed that physicians’ awareness about the risks of chronic BZD use was adequate, though their attitudes and self-perceived skills towards promoting BZD withdrawal could be improved. Interventions in primary care are needed to enable physicians to better motivate patients for BZD withdrawal.^35^ In Spain the analysis of the effectiveness of an intervention targeted to GPs to reduce BZD prescription and evaluate the implementation process has been published.^36^

## Conclusion

There is widespread prescribing of BZDs contrary to clinical guidelines by GPs in France, but the problem is worldwide. Prolonged or excessive prescribing is likely to cause harm (including death and addiction) and be an unnecessary cost to health services, particularly in vulnerable populations (such as older patients, young people, and patients receiving OST). Consequently, this is a major public health issue. This can be tackled through increased pharmacovigilance and addictovigilance, changes to prescriptions, and changing GP attitudes and prescribing behaviour.
